# In the Land of the Blue Dog*From Dr. Daniel’s *Recollections of a Rebel Surgeon*.

**Published:** 1910-11

**Authors:** Ferdinand Eugene Daniel


### Abstracts and Selections.

### In the Land of the Blue Dog.*

Said the Old Doctor, taking his usual seat:

Just after the war, when I was practicing medicine at Jackson, the capital of Mississippi, the home of my earlier days, I was requested by letter to go to one of the extreme eastern counties to see a case with a view to a surgical operation.

The eastern counties are, as I once told you, for the most part

*From Dr. Daniel's *Recollections of a Rebel Surgeon.*

piney woods, heavy sandy lands, no soil to speak of, except here and there where a creek or "branch" meanders through. These little creek bottoms, as they are called, afford at intervals little patches of tillable soil, and at long intervals you will come across a cabin, with its household of white-headed children, and a yellow dog—or a blue one most likely—and nearby a small clearing, fenced in by brush interwoven so as to turn even a rabbit, in which enclosure you will see a little crop of stunted yellow corn, or a patch of bumble-bee cotton.

"What is 'bumble-bee' cotton, Doctor?" said Hudson.

You are a greeny, sho nuff. Dan'els knows. It's cotton that a bumble bee can suck the top blossoms standing flat-footed on the ground, said the Old Doctor, nearly strangling, he laughed so hard at Hudson's unsophistication, and presently resumed his narrative.

The country is, of course, very sparsely settled off of the line of railroad, and mostly by the poorer classes—"tackeys," "po' white trash," the negroes call them. Now and then there is a more pretentious farm and a fairly well-to-do family; such a one as I was now on my way to visit. The stretches of pine trees and sand are interminable, and sometimes in a day's ride you will not see a living soul nor a sign of habitation; and they *do* say that when a jay bird or a crow has occasion to fly over, say Jasper county, for instance, if he is an experienced traveler or a close observer of events, or if he takes the papers, he always carries along a little sack of shelled corn.

In that section of country they have two or three names for a postoffice settlement; for instance, Damascus the natives call "Sebastopol"; Fairfield is "Bucksnort," etc. This I learned on the trip, as I will presently tell you.

Arriving at the nearest railroad station, I hired a double team, and getting my directions to Mr. Garrett's, near Damascus, I lit out for a thirty-mile ride, all by my lonesome. It was early fall, a gloomy day, the skies were overcast and the pines were soughing, as they do at the approach of rain. Oh! it's the lonesomest feeling imaginable. I rode and rode, mile after mile, through an unbroken monotony of those stately columns of long-leaf pine and sand. Not a living thing did I see except a buzzard, and he had evidently neglected to carry the essential bag of corn, and had fallen exhausted by the roadside before he had crossed the desert.

By and by, away towards sunset, my eyes were gladdened by the sight of a clearing. There was the little patch of stunted yellow corn, burnt up by the drought and the sun, and a little patch of bumble-bee cotton, and a rank growth of gourd vines on the fence of what had evidently been attempted for a vegetable garden and abandoned in despair. There had been a rail fence around the house once, but it was down and scattered; the yard was littered with paper and trash, and the house, which was a one-room log cabin, with a dirt-and-stick chimney, was closed and looked deserted. The lethean stillness, stirred—not broken—by the funereal soughing and sighing of the pines, dying away in the bosom of the interminable forest, like the wail of some lost spirit, was only accentuated by the rapping of a red-headed woodpecker on the sonorous boards of the gable. My heart sank within me. I thought I would make one effort, anyway, so I hailed:

"Hello!"

No reply.

"*Hello!!*" said I, louder.

Thereupon a blue and white hound dog, of the flop-eared species, crawled out from under the cabin, and putting all four feet together humped his back, gaped, protruding a long, pointed tongue, turned up at the end like a hook, yawned, thus giving himself a good stretch, lazily remarked:

"Brew-er-er-er-erh!"—something between a howl and a bark, curling it up at the end with a rising inflection on the last syllable.

"HELLO!!" said I again, louder.

The door opened and a strapping girl of about 16, perhaps, bare-legged to the knees, barefooted, with a dirty homespun dress on, came out on the porch, her yellow hair, cut off square all around, falling loosely on her neck.

"Can you tell me how far it is to Damascus, please?" I said.

"Wh-wh-i-c-h?" said she.

"How far is it to Damascus, please?"

"I kin tell you how far it is to the p-o-o-o-l!" she said, turning the "pool" up at the far end.

"What pool is it you are speaking of, Miss?" said I.

"They call it the *Sevas*terpool," said she.

"Well, how far is to Sebastopol, then?" said I, jumping at the conclusion that Sebastopol was the home name of Damascus, my place of destination.

"Hits about *fo'*-miles," said the girl. "You jes git inter ther road again, and keep on twell you git to the top of ther hill, and then you jes keep on twell you git to ther bottom of ther hill, and then you cross ther creek,' and then you keep ther *straight pool road* twell you *git* thar."

"Thank you, Miss," said I, and I drove on.

"Bre-w-er-er-erh!" howled the blue dog, and crawled back under the cabin grumbling at having had his nap interrupted.

I had gone not over three-quarters of a mile, I think, when I came to a log blacksmith shop on the side of the road, and a plank cabin about 10x12 feet—a country "store"—closed. The smith was sitting in his door smoking a corn-cob pipe, and looking very lonely, and well he might, for of all the God-forsaken, desolate wildernesses I ever saw that was the worst. It was near night, and a white hen and a red rooster had already retired for the night on the bed of a broken wagon, while two lean shoats were quarreling over the warm side of a litter pile against the end of the store. I said:

"My friend, can you tell me how much farther it is to Sebastopol?"

"This is hit," said the man, without rising or taking his pipe from his mouth.

"*Which* is 'it?'" said I.

"This," he said.

"Meaning—?" I said, glancing around.

"Yes; this shop and that store; that Ratliff's; he's got the chillunfever; hits the *post*office, too," said the man, with, I thought, a show of local pride.

Rejoiced that I was so near the end of my journey, I dismounted, stretched my legs, and made inquiry how to reach Mr. Garrett's, and in a little while was safely beneath that gentleman's hospitable roof.

---

On another occasion, Dr. Bob Horner, a classmate of mine, practicing at one of the railroad stations in East Mississippi, sent for me to meet him at his place and go with him in consultation to see a surgical case in the interior. You know, I had come out of the war with a considerable reputation with the home-folks of Mississippi as a surgeon, and Bob thought a good deal of my attainments, anyhow. Arriving at the station at an early hour, I was met by Dr. Bob with his spanking double team, and everything in readiness for the trip and the proposed operation.

We had to go about thirty miles, an all-day ride. Driving is tedious in that heavy white sand, and there are the same monotonous, interminable stretches of long-leaf pine. We had talked out, having kept up a pretty lively chatter up to and including our noon rest and lunch. The lunch consisted of two cans of cove oysters, two bottles of ale and some crackers.

At noon we unhitched our team by a clear little stream that crossed the road, gave the horses some feed and let them drink. Before opening up our lunch, Dr. Bob said:

"Hold on a moment, Doctor; there's white perch in this creek, and I'll catch some for our dinner."

I didn't argue the question with him; I supposed he knew what he was talking about. So Bob rigged up a line and hook which he took out of his clothes somewhere, and turning over a log secured some beetles or other bugs for bait, and going a little way up the creek was soon angling for perch, while I was making a fire, as he had requested me to do.

He was not gone over fifteen minutes, I should say, when he returned holding up for my inspection four beautiful speckled perch, each about ten inches long. They were the prettiest fish I ever saw, though I was accustomed to what they call white perch at Jackson. These were silver white, mottled with purplish blotches, and as the little stream was as clear as crystal and as cold as ice, you may imagine they were a delicious morsel. I said:

"How are you going to cook them, Bob?"

"Watch me," he said.

Raking away the sand in a clear nice place. he put some coals in the opening. Killing the fish by a blow on the back of the head, and opening them, removing the gills and entrails, and sprinkling on them some salt which he produced from a paper taken from his vest pocket, he wrapped the fish in several thicknesses of newspaper and thoroughly soaked the paper in the creek; then he laid them on the coals and covered them with hot ashes and coals on top of that. "When the paper burns they are done," said Bob.

Meantime he had taken out the lunch, and spreading the laprobe on the ground for a table cloth, we spread our feast; and I tell you now I never in my life tasted anything that met my demands better than those white perch Bob roasted in the ashes.

We resumed our journey, and by 4 o'clock the horses were much jaded, and we had to take it slowly. We soon relapsed into silence, each one busy with his own thoughts; it was awfully "bore-ous.'

Presently, at the bottom of one of those long red hills that characterize a portion of that section, though for the most part the land is level, we came upon a covered wagon drawn by two lean ponies, and filled with white-headed children. Under the wagon a tar bucket hung loosely, and by it was tied a blue dog of the genus "hound." Out by the roadside lay a larger yellow and white dog,—dead. An old man with long gray beard was standing by, doing nothin' but lookin' sorry; a typical specimen of the "mover class," or, as Dr. Willis King in "Stories of a Country Doctor," calls them, "branch-water men." The old man had evidently just dragged the dog there and left him. By the man stood a tow-headed boy in butternut-dyed jeans pants, a coarse cotton shirt, and galluses of striped bed-ticking, with his hands stuck in his pockets up to his elbows, for it was a little coolish.

The scene was so desolate, the old man looked so sad, I thought to say a cheering word and perhaps get him into conversation; I didn't, of course, know what killed the dog; so in the absence of anything better to begin with, I sang out cheerily:

"My friend, did your dog die?"

He looked at me sorter sideways for about a minit. "I *reckin* so, by G-d—he's dead," said he with a scowl and a look as if he'd like to cut my throat for a darned fool.

Dr. Bob knocked me on the back and just "ha-ha'd." "A good one on *you*, Doctor," he said. "Now, don't you wish you hadn't said anything?"

"I do indeed," I said, much disgusted.

Bob said that class resent anything of the kind, and that it is best to speak to them when spoken to. I told him that I had just been told as much by the "other fellow."

Bob called my attention to the fact—he says it is a fact—that this class is as much characterized by the blue dog as the negro is by the "yaller" dog; and that the blue dog is found nowhere else than in the piney woods among the "poor folks," as they are universally called by the darkies.

But Dr. Bob's time came soon, said the Old Doctor. Just before dark—the chickens were flying up—we came in front of a nice white house, a Mr. Gregory's, a pretty well-to-do farmer. The house sits back from the road some little distance in a pretty lawn, surrounded by a neat white fence—evidences everywhere of thrift, contrasting strikingly with the absence of it almost everywhere else, and with the desolateness of the surroundings generally. Bob said:

"Here, Doctor, hold the reins; I've got to give these horses some water; they looked fagged out, and we have eight miles to go yet."

Just then a great big black dog—a fierce looking fellow—got up and gave a low growl.

"I'm awfully afraid to go in there; that's a terrible dog. I know this country from one end to the other, and I've heard of Dave Gregory's dog."

"Here boy," said the doctor to a lad standing near the dog. "If you'll hold that dog till I get two buckets of water, I'll give you a quarter."

"All right," said the boy, and he seized the dog around the neck. "Come ahead," said he, "I'll hold him," and he pushed the dog to the ground, and with his arm around him laid down on top of him.

The doctor, taking the bucket from the foot of the buggy in one hand, and the heavy driving whip in the other, holding it by the small end, ready to use it as a club if necessary for defense, went cautiously in, circling around the dog and keeping a sharp eye on him.

He got the water and watered both horses; and just before getting into the buggy said:

"Boy—don't turn that dog loose till we get started—and here's your quarter on the gate post."

"All right," said the boy; "down, sir" (to the dog).

As Bob got into the buggy and took hold of the reins he said:

"That's a pretty savage dog, ain't he, Bud?"

"He *uster be*," said the boy.

"Use to be?" said the doctor; "ain't he bad now? Won't he bite?"

"Bite nothin'," said the boy, pocketing the quarter. "He's b-b-b-blind, and so old his teefs is all dropped out."

"One on you now, Doc," said I. "Don't you wish you had your quarter back?"

FERDINAND EUGENE DANIEL.

